# A Novel GLP-1 and FGF21 Fusion Protein for the Treatment of Non-alcoholic Steatohepatitis (NASH)

**DOI:** 10.34172/apb.43672

**Published:** 2024-12-10

**Authors:** Zhipeng Zhang, Yanqin Ma, Cheng Xie, Yan He, Dong Wang, Huaien Song, Miao Yuan, Xiaomei Zhang

**Affiliations:** ^1^Laboratory of Pharmaceutical Engineering, School of Life Science and Health Engineering, Jiangnan University, Jiangnan University, Wuxi, China; ^2^Suzhou Hepa Thera Biopharmaceutical Co., Ltd., Shanghai, China; ^3^WuXi AppTec (Shanghai) Ltd., China

**Keywords:** GLP-1, FGF21, NASH, Fusion protein, Weight loss and fat reduction

## Abstract

**Purpose:**

The objective of this study was to develop and produce a novel fusion protein that combines GLP-1 (glucagon-like peptide-1) and FGF21 (fibroblast growth factor 21), with the aim of achieving synergistic pharmacological effects through the targeting of dual pathways, followed by validation of these effects in a non-alcoholic steatohepatitis (NASH) model.

**Methods:**

We utilized C57Bl/6J mice to establish a high-fat diet (HFD)/CCl_4_ NASH model, with the aim of assessing the drug efficacy across low, medium, and high (0.3, 1, 3 mpk) dose groups administered twice a week for 28 days.

**Results:**

The animal pharmacological experiment of HSP763-01 demonstrated a significant reduction in body weight without apparent appetite suppression. Analysis of blood biochemical indicators revealed a marked decrease in triglycerides (TG), serum total cholesterol (TCHO or TC), low-density lipoprotein (LDL), and blood sugar levels with a significant dose-dependent effect. Additionally, Liver tissue analysis indicated notable alleviation of liver fatty degeneration and ballooning degeneration, as well as partial relief of lobular inflammation with a significant dose-dependent effect. However, due to the severe liver fibrosis induced by tetrachloromethane (CCl_4_) in mice (3rd grade), HSP763-01 exhibited limited efficacy in alleviating fibrosis.

**Conclusion:**

HSP763-01 exhibited a clear dual target of GLP-1 and FGF21, which has been demonstrated by a robust response to the HFD/CCl_4_ model, showing a marked improvement in lipid metabolism, lowering of blood glucose, weight loss, significant alleviation of liver steatosis, ballooning, and partial relief of lobular inflammation and fibrosis.

## Introduction

 Non-alcoholic fatty liver disease (NAFLD) is one of the main causes of liver disease worldwide, with an estimated prevalence of around 25%, highest in the Middle East and South America, and lowest in Africa,^1^ Simple fatty liver, clinically referred to as NAFLD, is distinguished from the more severe condition characterized by inflammation and hepatocellular injury, known as non-alcoholic steatohepatitis (NASH). The incidence of NASH is on the rise, and the disability and mortality rates of NASH patients are significantly higher than those of NAFLD patients.^2^ NASH causes irreversible damage to the body, and if not treated in a timely manner, it can progress to liver cirrhosis and liver cancer. Recent reports have indicated that the rate of liver cirrhosis in NASH patients has reached 16%,^3^ There are also reports of a high recurrence rate of NASH after liver transplantation, and the mortality rate of NASH patients within one year after transplantation is also generally higher. In general, NASH is accompanied by intercellular fibrosis, which may progress to liver cirrhosis.^4,5^ Several drugs have entered the clinical trial stage, and clinical studies have demonstrated their efficacy in reducing liver fat, improving inflammation, and fibrosis. AP026, a FGF21 (fibroblast growth factor 21)/ GLP-1 (glucagon-like peptide-1) bifunctional protein, is currently undergoing phase 1 clinical development for the treatment of NASH and type 2 diabetes mellitus (T2DM). HEC88473 is a bi-specific fusion protein of GLP-1/FGF21 that has already been clinically used to manage blood glucose control, weight loss, and NASH in patients with type 2 diabetes. It is currently in phase 2 clinical trials. DR10624 is a triple activation Fc fusion protein targeting GLP-1/glucagon receptor (GCG) /FGF21 that exhibits synergistic effects. It is currently in phase 1 clinical trials and has shown significant improvements in lipid levels, weight management, and glucose control. In terms of treatment, it’s worth noting that the world’s first new drug for NASH was approved for marketing in the United States on March 14th this year. Resmetirom, it has demonstrated efficacy in reducing hepatic fat content, improving liver histology (both NASH resolution and fibrosis improvement), and ameliorating biomarkers of liver damage without significant effects on body weight or glucose metabolism.^6^ The US FDA officially approved resmetirom for the treatment of NASH patients with liver fibrosis.^6-9^

 GLP-1 is a 30-amino acid peptide hormone produced in the intestinal epithelial endocrine L-cells by differential processing of proglucagon, the gene which is expressed in these cells.^10^ Its receptors are widely distributed in the nervous system, pancreas, heart, lungs, and skin, among other organs,^11^ GLP-1 also reduces gastric emptying and food intake, thereby enhancing nutrient absorption while minimizing weight gain.^12^ The evolution of GLP-1 receptor agonists (GLP-1RAs) has progressed from short-acting formulations to long-acting and subsequently ultra-long-acting agents, including exendin-4, liraglutide, semaglutide, and the ultra-long-acting dulaglutide.^13^

 FGF21 is an effective regulator of glucose and lipid metabolism, exerting its biological effects through FGF receptors (FGFRs) and β-klotho,^14^ FGFRs, which include FGFR1, FGFR2, FGFR3, and FGFR4 are transmembrane proteins. FGF21 is required to increase its affinity to the β-klotho receptor. The natural forms of FGF21 can be rapidly cleared through the kidneys due to their low molecular weight (approximately 19.4 kDa). To increase their molecular weight, prolong circulating half-life, and prolong drug exposure, one common method is fusing PEG (polyethylene glycol) fragments to the recombinant protein. Another method is to coupling it with IgG molecules, ultimately reducing liver fat and liver cell damage, while simultaneously inhibiting inflammation and fibrosis.^15-17^

 Obeticholic acid (OCA [6-ethyl-chenodeoxycholic acid]) is a semi-synthetic bile acid analogue with binding affinity to the farnesoid X receptor (FXR), OCA attenuates body weight gain and improves fatty liver pathology in humans and mice,^18^ but the therapeutic effects of OCA in NASH are at best modest, despite improvement in steatosis, there is not reversal of NASH pathology,^19^ however, OCA treatment was associated with an increase in LDL-C and total cholesterol which was largely due to an increase in less-atherogenic small VLDL and large-buoyant LDL particles.^20^ OCA reduced liver weight and lipid in NASH mice (both by 17%) but had no effect on plasma ALT or AST levels.^21^ OCA was shown to improve fibrosis in patients with NASH in the clinical trial, In the Phase 2b Farnesoid X Receptor Ligand Obeticholic Acid in Nonalcoholic Steatohepatitis Treatment trial, histologic features, including fibrosis stage, improved in significantly more patients treated with OCA than with placebo.^22^ Considering that OCA is in the late stages of clinical development and possesses extensive data regarding its efficacy in treating NASH, we have chosen it as a positive control drug for our preclinical pharmacological studies to validate the reliability of the NASH model and to provide a benchmark for assessing the efficacy of our novel fusion protein.

 Recent studies have reported that the combination of GLP-1 and FGF21 has a synergistic effect on blood glucose control.^23^ Increasing evidence suggests that GLP-1, when combined with FGF-21, plays crucial roles in maintaining glucose and lipid homeostasis in metabolic disorders.^24^ The weight loss caused by GLP-1 agonists is believed to be partly due to the increased plasma FGF-21 levels induced by glucagon signaling,^25^ fusing FGF21 with GLP-1 would provide the thermogenic benefits to complement reduced food intake for maximal weight reduction, without diminishing the glucose control afforded by GLP-1. Furthermore, rather than localizing glycemic effects to the pancreas—as is the case with members of the glucagon superfamily—the FGF21 component has the potential to further improve glucose control by acting on peripheral tissues to increase insulin sensitivity.^23,26^ However, co-administration of drugs not only increases the frequency of drug administration for patients, but also reduces their compliance with treatment, on the other hand, it will greatly increase the cost of treatment. In addition, there have been reports of preparing dual-function proteins by fusing GLP-1 and FGF21, GLP-1-Fc-FGF21 has shown effective and sustained glucose control at much lower doses in diabetes mouse models, and superior weight loss, lipid distribution improvement, and anti-NASH effects in high-fat diet (HFD)-induced ob/ob models.^27^ We hypothesize that the newly developed dual-target GLP-1/FGF21 fusion protein HSP763-01 will provide superior efficacy in weight loss, glucose control and anti-fibrotic effects compared to single-target treatments, addressing both the metabolic and inflammatory aspects of NASH in the HFD/CCl_4_ mouse model.

## Method and Materials

###  Animals

All animal care and experimental procedures are conducted in strict accordance with internationally recognized principles for the ethical use and welfare of laboratory animals. Our research institution’s animal facilities and Institutional Animal Care and Use Committee (IACUC) are accredited by the Association for Assessment and Accreditation of Laboratory Animal Care International (AAALAC). All aspects of animal husbandry, management, euthanasia, and experimental procedures will adhere to AAALAC-related standards and principles. The study utilizes male C57BL/6J mice at approximately 19 weeks old, as well as Diet-Induced Obese (DIO) mice weighing over 40 g, provided by GemPharmatech Co., Ltd. A total of 56 DIO mice and 8 normal mice are required for this study. Upon arrival at the laboratory, designated personnel will monitor their health status before randomly selecting a healthy control mouse for testing against specified pathogens (*Staphylococcus aureus*, *Pseudomonas aeruginosa, Trypanosoma cruzi, Helicobacter hepaticus*, and mouse hepatitis virus). Following their arrival at the laboratory, all mice will undergo 13 days acclimation period, high-fat diet was provided while normal mice receive a conventional diet. Environmental conditions will be maintained at a temperature range of 20-25 °C with relative humidity between 40%-70%, under a light cycle of 12 hours per day (7:00-19:00). The mice will have ad libitum access to feed and water.

###  Preparation of model and study design

 In the preliminary phase of this experiment, DIO mice were induced by feeding them with a high-fat diet, and then in the subsequent phase. CCl_4_, coupled with a high-fat diet, stimulates an increase in hepatic triglycerides (TG) and plasmatic cholesterol, as well as collagen deposition in the liver and hepatic transaminases in the plasma,^28^ therefore, a NASH model was induced by injecting CCl_4_ into the peritoneal cavity while feeding them with a high-fat diet, and evaluating the anti-NASH effectiveness of the test compound in this model. Key Instruments (Model): Biosafety Cabinet (BSC-IIA2 1.5m), Low-Temperature Centrifuge (Thermo-Fresco 17), Analytical Balance (CPA225D). After the animals arriving at the facility, a healthy animal was selected for testing, while the others were provided with either high-fat or normal feed. The high-fat feed was refreshed daily, and the acclimation period lasted for 13 days. Throughout this period, the healthy status of the animals was monitored daily. Any abnormalities or infections discovered will result in exclusion from the experimental groups. On day -1, DIO mice were randomly allocated into groups to minimize inter-group differences, with 8 mice in each group and 8 healthy control mice in group 1. CCl_4_ was induced by thorough shaking before mixing 1 part of CCl_4_ with 3 parts of olive oil in a glass bottle to create a 25% CCl_4_ solution and used immediately; any unused solution should be disposed (The preparation process should take place within a biological safety cabinet and minimize exposure time). Animals in groups 2-7 received intraperitoneal injections of CCl_4_ twice weekly for a total of 8 times; those in group 8 received intraperitoneal injections once a week for 6 times; while the normal healthy group (group 1) received saline via intraperitoneal injections twice a week for 8 times. The dosage of both the 25% CCl_4_ and saline solutions was based on each animal’s weight at 0.5 mL/kg.

**Figure 1:**
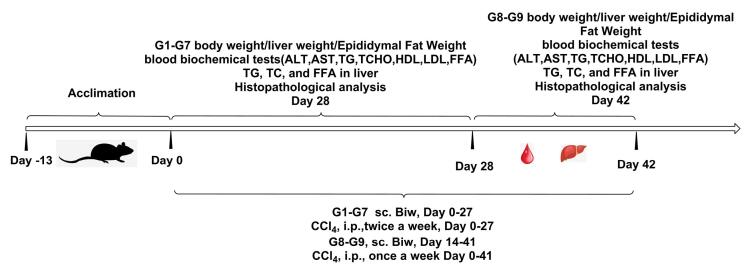
Construction and design of NASH HFD/CCl_4_ model

 The injection time of CCl_4_ should be at least 4 hours apart from the administration time point on the same day. The first injection of CCl_4_ to animals was set as Day 0, and the NASH model was constructed by administering HFD + CCl_4_. The dosing regimen was designed into 9 groups, with 8 animals in each group, including a control group that received (Deoxycholic acid) OCA once a day at a dose of 1% (hydroxypropyl methyl cellulose) HPMC, 30 mg/kg; the HSP763-01 fusion protein with dual targets of GLP-1 and FGF21 was prepared in normal saline, and the dose-related study was conducted at low dose group (0.3 mg/kg), middle dose group (1 mg/kg), and high dose group (3 mg/kg); additionally, group 7 examined the stability of HSP763-01 GF6 (1 mg/kg) at -20 °C for about 30M, and compared the pharmacological effects with group 5; group 8 examined the pharmacological effects of CCl_4_ pre-model induction for 2 weeks followed by drug administration for 4 weeks, and compared with group 9, the model control group. The overall experiment results were compared with NASH model control groups, as well as the positive control group of OCA, and the blank control group. The specific method of constructing the NASH model and the dosing regimen design were shown in Figure 1 and Table S1 (Supplementary file 1).

###  Analysis of body and liver weight changes

**Figure 2:**
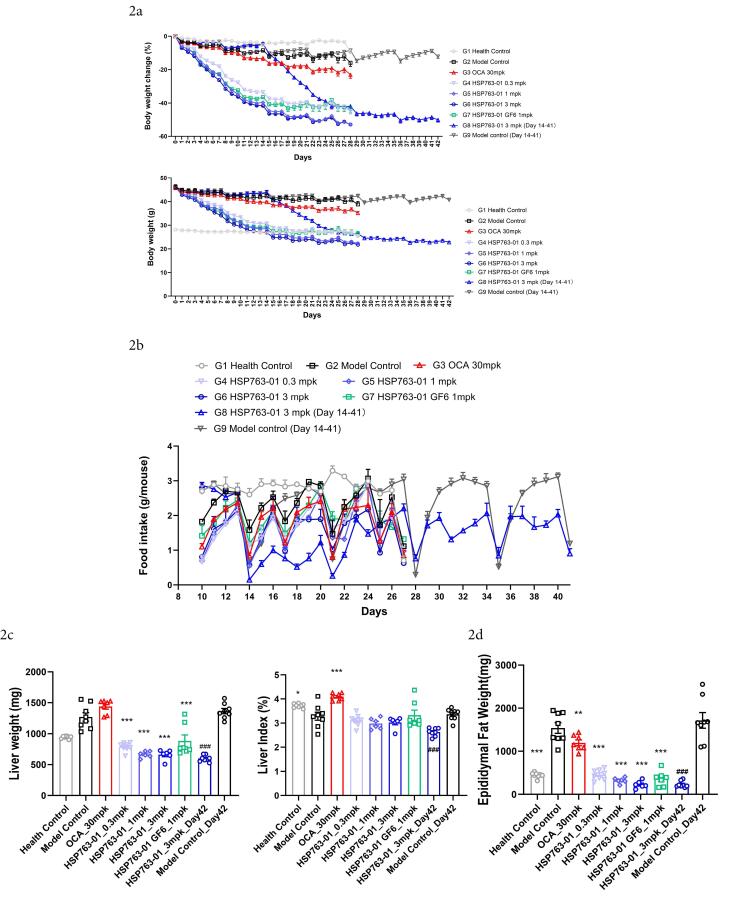
(2a) Development of body weight during the intervention period. N = 8 per group, Data were presented as Mean ±SEM; (2b) Food intake throughout the duration of the study. N = 8 per group, Data were presented as Mean ±SEM; (2c-2d) At the end of the study, analyses were performed to evaluate liver weight, liver index, and epididymal fat weight, Data were presented as Mean ±SEM. ***P<0.001, **P<0.01, *P<0.05 vs model control and healthy control, analyzed by one-way ANOVA. Health Control vs Model Control Day 42, analyzed by t-test ###*P*<0.001, ##*P*<<.01, #*P*<0.05

 From group 1 to group 9, the first injection of CCl_4_ was set as day 0, and the mice were weighed daily and compared with the healthy control group, see Figure 2a for details. Start recording daily diet from Day 10 and comparing the results among different groups for analysis, as shown in Figure 2b. For the analysis of liver weight, each experimental group was subjected to terminal dissection, where the animal liver was collected (Day 28, Day 42), washed with physiological saline, then dried with sterilized tissue paper, weighed, and recorded, as shown in Figure 2c. The weight of the terminal epididymal fat was collected (Day 28, Day 42), weighted and transferred to -80°C storage afterward (Figure 2d). The HSP763-01 treatment groups, OCA treatment group, and healthy control group were studied for 28 days. The HSP763-01 treatment group showed a more significant weight loss compared to the model control and healthy control groups, and there was no significant further weight loss when the treatment was continued after the weight reached that of the healthy control group, showing a clear dose-dependent effect. Group 8 and group 9 underwent a 42 days observation and research, with CCl_4_ serving as a model to induce for 2 weeks, followed by a 4 weeks administration of HSP763-01, which resulted in a significant weight loss to the level of the healthy group and then maintained at that level. The doses of the drug did not affect the amount of food consumed by mice, while injection of CCl_4_ was irritating and affected the amount of food consumed on that day, but gradually returned to normal. Compared with the model control group, the liver weight and epididymal fat level of the treated mice were significantly lower (*P* < 0.001), indicating that HSP763-01 has the function of reducing body fat synthesis and promoting fat metabolism utilization, which can be applied to the treatment of obesity and metabolic syndrome caused by obesity.

###  Blood biochemical analysis

 The detection of serum markers was performed according to the instructions of the commercial kits, processing the serum samples by Biochemical Analyzer (Bechman AU480), aspartate aminotransferase (AST) kit (Bechman OSR6109), alanine aminotransferase (ALT) test kit (Bechman OSR6107), cholesterol testing kit (Bechman OSR6116), triglyceride test kit (Bechman OSR6118), low-density lipoprotein (LDL) cholesterol test kit (Bechman OSR6183), High-density lipoprotein cholesterol Test Kit (Bechman OSR6187).

 The detection of triglyceride (TG), total cholesterol (TC), free fatty acid (FFA) in liver tissue was performed according to the instructions of the kit. The TG quantification kit was provided by Nanjing Jiancheng Bioengineering Research Institute Co., Ltd. (Lot No. 20220903); the cholesterol quantification kit was provided by Nanjing Jiancheng Bioengineering Research Institute Co., Ltd. (Lot No. 20220808); and the free fatty acid assay kit was provided by Nanjing Jiancheng Bioengineering Research Institute Co., Ltd. (Lot No. 20220914).

 According to the administration schedule, group 1 to group 9 underwent a complete fast for one day. Starting from day 28 (group 1 to group 7), and day 42 (group 8, group 9), anesthesia was administered for blood collection and the measurement of blood glucose as well as biochemical indicators (ALT, AST, TG, TC, HDL, LDL-C, FFA). Tissue samples were collected at the experimental endpoint (day 28, day 42). After terminal sampling was conducted, blood was collected from the heart after anesthesia, the neck was clamped to confirm death, and as much blood as possible was collected. Serum was also collected and divided into two separate centrifuge tubes (serum collection method: 10 000 rpm, centrifuged at 4 °C for 10 minutes, collected serum, transferred to dry ice and stored at -80 °C). Collected animal livers, rinsed with physiological saline; blotted with sterilized tissue paper, weighed and recorded; taken liver tissue from the left, middle, and right lobes and immersed them in 10% formalin solution overnight for fixation. Frozen livers and epididymal fat were collected and transferred to -80 °C storage.

###  Histopathological analysis

 In the mouse groups corresponding to Day 28 and Day 42, the left, middle, and right lobes of the liver were collected at room temperature and fixed in 10% neutral buffered formalin overnight for histopathological analysis. The liver tissue was processed for paraffin embedding in an automated dehydration machine (Leica HistoCore Pearl) using the formalin-fixed tissue dehydration protocol. Through hematoxylin & eosin (HE) staining, the tissue sections were stained to varying degrees of red or pink. The transparent tissue sections could be clearly reflected by HE staining, showing the organization structure, tissue type, cell layer, etc. It was possible to clearly observe the general morphological and structural features of various tissues or cell components and pathological changes under a microscope. The NAS score was given to the HE-stained sections, which consists of fatty degeneration, ballooning degeneration, and lobular inflammation. Sirius Red staining was used to identify collagen fiber networks in tissue sections, qualitatively identifying abnormal changes in the collagen network in degenerative diseases, hereditary or acquired disorders, and using morphological imaging analysis.^29^ This experiment was analyzed by the fibrosis changes in the liver tissue sections and scored. All Sirius Red-stained whole slides were scanned using the Leica Aperio AT2 Brightfield scanner, and then the percentage of Sirius Red positive staining area was calculated by the HALO AI system to assess the percentage of Sirius Red area on the total scanned liver area. The automated immunohistochemistry workflow based on HALO utilizes a series of pre-set AI algorithm models to automate the quantification of positive cells, aiming to improve the efficiency and accuracy of immunohistochemistry quantitative analysis. The deep learning system of HALO AI integrates multiple neural network technologies seamlessly into the digital pathology platform HALO system. Stain OD (Weak, Moderate, Strong) - For each stain identified by the specification of thresholds that define negativity and three levels of positivity (weak, moderate, and strong) for the corresponding stain. Pixels with OD values below this threshold are considered negative for the Stain. Pixels with intensities greater than or equal to this threshold, but also less than the moderate OD threshold are considered weakly positive. Pixels with OD values greater than or equal to this threshold, but also less than the strong OD threshold are considered moderately positive. Pixels with OD values greater than or equal to this strong threshold are considered strongly positive.

###  Molecular design

 Dulaglutide is a fusion protein of GLP-1 and the Fc domain of immunoglobulin G (IgG4), which exhibits prolonged pharmacokinetics and activity. Through engineering modifications, it has significantly improved plasma half-life (approximately 30 hours) and reduced clearance rate.^30,31^ This study engineered and refined the original sequences of GLP-1 and FGF21. The GLP-1 sequence remained consistent with the sequence of Dulaglutide, the amino acid sequence of GLP-1 including the linker was as follows: HG (Alanine A was partially resistant to enzymatic degradation by DPP-IV due to its substitution by glycine G) EGTFTSDVSSYLEE (Glycine G was replaced by glutamate E) QAAKEFIAWLVKGGGGGGGSGGGGSGGGGSA. It was worth noting that Ala was replaced by Gly in human GLP-1 sequences,^32^ this modification was conferred to partial resistance to DPP-IV enzymatic degradation, thereby extending its half-life in the body and preventing degradation by DPP-4 enzyme.^33,34^ Based on the fusion of GLP-1 sequence with the IgG4 Fc domain of the immunoglobulin,^34,35^ we have fused GLP-1-IgG4 Fc (The amino acid sequence with linker was as follows: HGEGTFTSDVSSYLEEQAAKEFIAWLVKGGG GGGGSGGGGSGGGGSAESKYGPPCPPCPAPEAAGG PSVFLFPPKPKDTLMISRTPEVTCVVVDVSQED PEVQFNWYVDGVEVHNAKTKPREEQFNSTYRV VSVLTVLHQDWLNGKEYKCKVSNKGLPSSIEK TISKAKGQPREPQVYTLPPSQEEMTKNQVSLTCLVK GFYPSDIAVEWESNGQPENNYKTTPPVLDSDGSFF LYSRLTVDKSRWQEGNVFSCSVMHEALHNHYTQK SLSLSLG) with a mutant form of FGF21 to achieve further extending the in vivo half-life and increasing the dual targeting effect.

**Figure 3:**
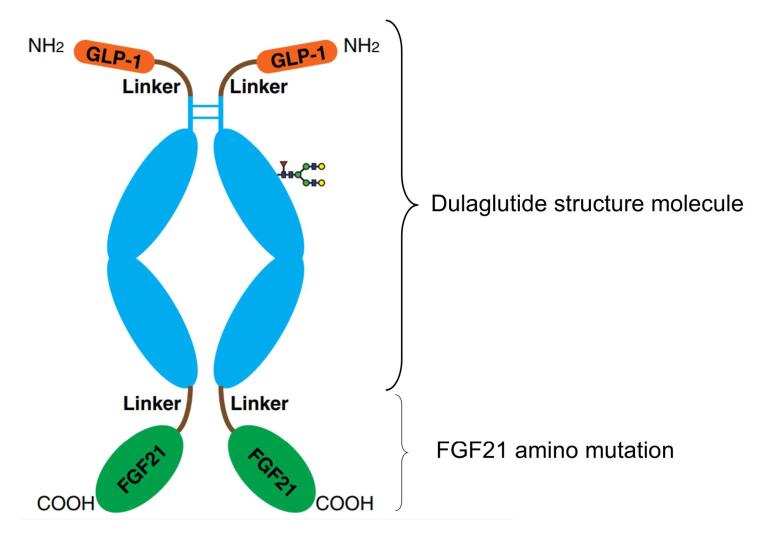
HSP763-01 structure molecule

 Efruxifermin^36^ is a long-acting Fc fusion protein consisting of two modified human fibroblast growth factor FGF21 variants, which are linked via a short glutamine-serine linker sequence to the human IgG1 Fc domain. Compared to the native human FGF 21, the following point mutations in the FGF21 portion of efruxifermin enhance its biological or pharmacological activity: L98R reduces the tendency to aggregate; P171G restricts the endogenous endopeptidase and fibroblast activation protein (FAP) from degrading the C-terminus of efruxifermin; A180E increases the affinity for the FGF21 co-receptor β-klotho. These modifications enhance the in vivo pharmacodynamic activity of efruxifermin while extending its duration of action.^37-39^ The amino acid sequence of the Human FGF21 Recombinant Protein studied in this research was as follows: (DSSPLLQFGGQVRQX_15_YLYTD DAQQTEAHLEIRE DGTVGGAADQSPESLLQLKALK PGVIQILGVKTSRFLCQRPDGALYGSLHFDPEACS FREX_94_LLEDGYNVYQSEAHGLPLHX_114_PGNKSPHRD PAPRGPX_130_RFLPLPGLPPALPEPPGILAPQPPDVGSS DPLSMVGGSQGRSPSYX_176_S), We selected the following mutations at the X_15_, X_94_, X_114_, X_130_, and X_176_ sites, corresponding to V, R, L, A, and E respectively. The findings were presented in Figure 3.

###  Construction of fusion protein expression plasmids

**Figure 4:**
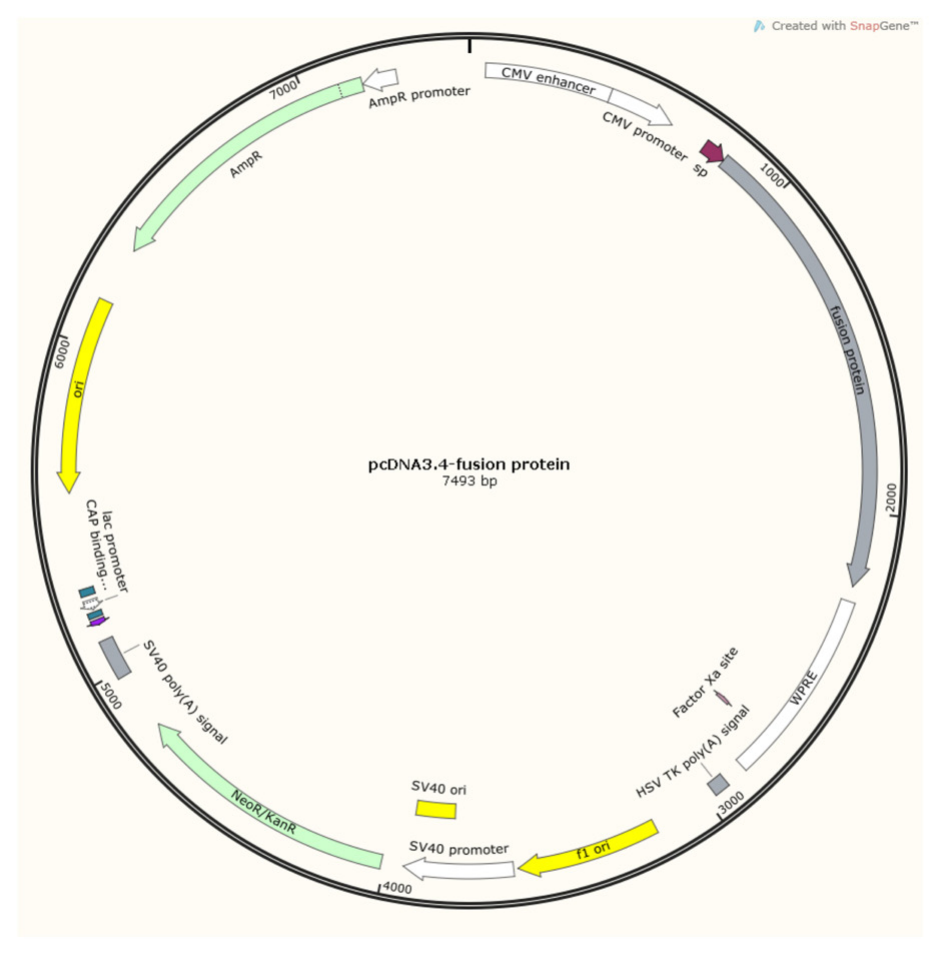
pcDNA3.4-fusion protein. (Software: SnapGeneTM1.1.3)

 To prepare a plasmid expressing HSP763-01, we commissioned Azenta Life Sciences to synthesize it. Azenta optimized (for Cricetulus griseus CHO) and routinely synthesized the targe gene, and cloned the gene into the vector pcDNA3.4( + ) (Ampicillin) (Invitrogen, A14697) via XbaI (Takara, 1093A) and AgeI (ABonal, RK21125). The resulting plasmid was named pcDNA3.4-fusion protein (Figure 4). Kits from MACHEREY-NAGEL were used to purify endotoxin-free plasmid DNA.

###  Transfection of cells expressing fusion protein

 For transfection, CHO K1 cell stock (provided by Haoyang Biotech from ECACC Global Sub-Licensed) was used along with PEI transfection reagent. Following transfection into CHO cells and a 10-day culture period, cell supernatant was harvested and analyzed using ProA-HPLC. The transient transfection expression level after 10 days of cell culture was determined to be 288.74 mg/L.

###  Protein purification and analysis

**Figure 5:**
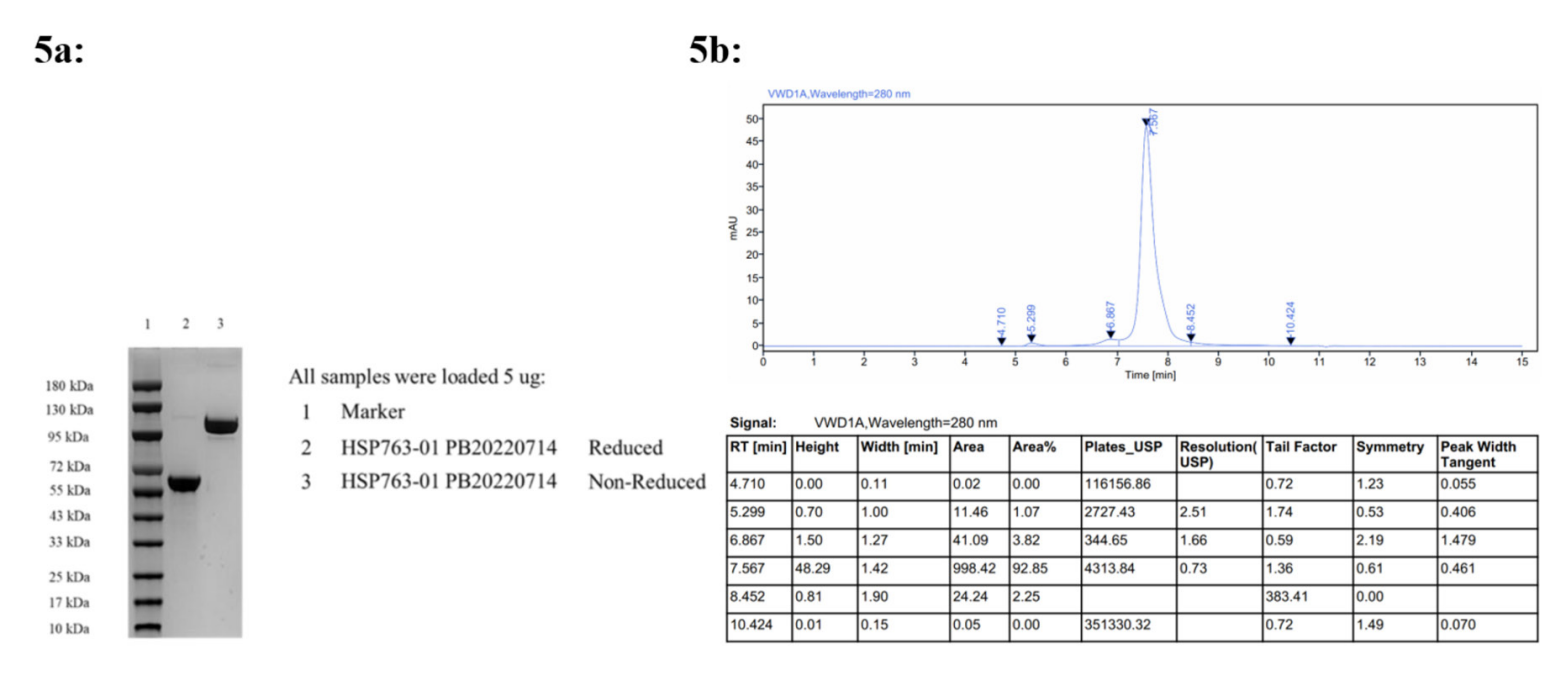
5a, SDS-PAGE Purity Analysis; 5b, SEC-HPLC Purity Analysis 92.85 %

 Antibody purification was as follows, Affinity chromatography: Buffer solution- Buffer A: 1 × PBS, pH 7.4; Washing solution- Buffer B: 25mM Sodium citrate, pH 3.0. Centrifuge the cell culture medium at 10 000 g for 15 minutes, followed by filtration through a sterile 0.22 μm filter. Subsequently, equilibrate the chromatography column with buffer A by flushing it with approximately 5 column volumes. Employ a linear gradient elution method to isolate the protein and collect the fractions in separate tubes. Further purify the protein using a cation exchange chromatography column. Achieve baseline equilibrium of the column by balancing it with 5 column volumes of Buffer A, then flush again with Buffer A for about another 5 column volumes before performing elution using a linear gradient method with buffer B. Collect and analyze each fraction’s purity using appropriate analytical techniques. Select those fractions that meet purity criteria and combine protein samples from different tubes accordingly. Finally, concentrate the pooled protein sample and exchange its buffer to PBS at pH 7.0. The HSP763-01 fractions were characterized by SDS-PAGE and SEC-HPLC, Table S2 (Supplementary file 1), sterile-filtered (0.22µm), assessed for concentration (absorption at 280 nm) and stored at -20 ^◦^C. HSP763-01 Protein Preservation Solution: 20 mM CA (0.02% T-80 + 5% Mannitol), pH 7.0. Concentration 1.04mg/mL, the specific quality control results were shown in Figure 5.

## Results

###  Blood biochemical analysis

**Figure 6:**
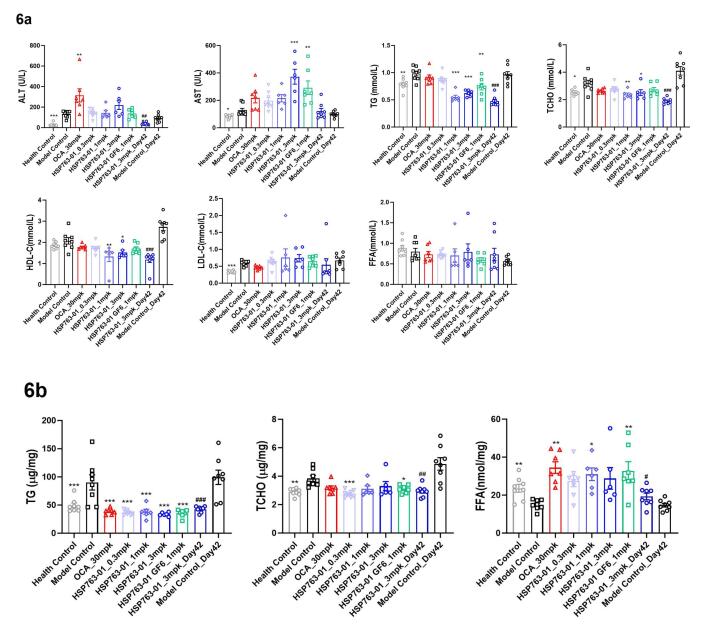
(6a). Graph of blood biochemical indicators measured at the endpoint for group 1 to 9; (6b). Graph of biochemical indicators measured at the endpoint for liver tissue sampling in group 1 to 9. N=8 per group, N=8 for vehicle control group and model control group, Data were presented as mean±SEM. ***P<0.001, ** *P*<0.01, * *P*<0.05 vs NASH Control, analyzed by one-way ANOVA. Health Control vs NASH Control, analyzed by t-test. ### *P*<0.001, ## *P*<0.01, # *P*<0.05 vs model control Day 42, analyzed by t-test

 The results of the biochemical tests were shown in Figure 6a. TG, TC, and FFA were tested on liver tissue, as shown in Figure 6b. From the results of blood biochemistry, compared with the model group, HSP763-01 at 1 mpk and 3 mpk could significantly lower TG and TC levels (*P* < 0.001), while these indicators were also mild lowered at 0.3 mpk dose, but without statistical significance. Among them, HLC-C slightly decreased to the normal group level (*P* < 0.01, *P* < 0.05), and LDL-C slightly fluctuated among individual mouse in different groups, moreover, blood glucose levels were significantly reduced to within the healthy range (*P* < 0.001), and even lower, in all dosage groups, demonstrating a clear dose-dependent effect. From the results of liver biochemical indicators, compared with the model group, HSP763-01 at all doses (0.3 mpk, 1 mpk, 3 mpk) could significantly lower the levels of liver TG and TC (*P* < 0.001). HSP763-01 GF6_1mpk demonstrated significant decreases in serum biochemistry and liver triglyceride (TG) detection (*P* < 0.01, *P* < 0.001). However, no statistically significant difference was observed in serum biochemical total cholesterol (TC) detection, while a moderate reduction in liver TC detection was noted (*P* < 0.05). Except for serum biochemical TG detection, no significant differences were observed between HSP763-01 GF6_1mpk (stored at -20°C for 30 months) and newly prepared HSP763-01_1mpk; Compared with model control and healthy control groups, the level of liver FFA was increased in all groups, corresponding to the decrease of FFA level in the blood. Compared with the health control group, there was an increase in serum biochemical indicators ALT and AST at Day 28 and Day 42 in the CCl_4_-treated groups, suggesting that the CCl_4_ injection into the peritoneal cavity of mice has a stimulating effect on the liver. The stimulating effect of weekly twice intraperitoneal injection of CCl_4_ in the treatment group and the model control group was significantly higher than that of weekly single intraperitoneal injection of CCl_4_ groups. Compared with the model control group, the detection of liver TG by HSP763-01 showed a very significant reduction from 0.3mpk, 1mpk, and 3mpk, consistent with the OCA positive control group and reaching the level of the healthy control group(*P* < 0.001); the detection of liver TCHO also showed a certain degree of reduction relative to the model control group and reached the level of the healthy control group (*P* < 0.001, *P* < 0.01, *P* < 0.05). The reduction of liver TG and TCHO was not significantly related to the dose relationship, which may be because the low dose of 0.3 mpk has already reached the level of the healthy group. Based on the blood biochemistry results, the HSP763-01 treatment group demonstrated a significant reduction in ALT, TG, TCHO, and blood glucose levels compared to the positive OCA group. However, it is important to note that within the HSP763-01 treatment groups at different doses, individual variability resulted in similar efficacy for lowering serum AST, LDL-C, and FFA levels, as well as liver FFA, TG, and TC levels.

###  Histopathological analysis

 NAS Semi-quantitative Scoring of HE-stained slides,^40^ The score consists of steatosis, ballooning, and lobular inflammation. The pathologist scores the slides according to the standards shown in Table S3 (Supplementary file 1).

 Analyzing the fibrosis changes in the tissue sections stained with Sirus-red staining. The fibrosis in each part was scored according to the standard indicated in Table S4 (Supplementary file 1).^41,42^ All paraffin-embedded sections were scanned using the Leica Aperio AT2 Brightfield scanner, and then the HALO AI system was used to calculate the percentage area of positive staining for Sirus-red to evaluate the percentage area of Sirus-red staining on the total scanned liver area.

**Figure 7:**
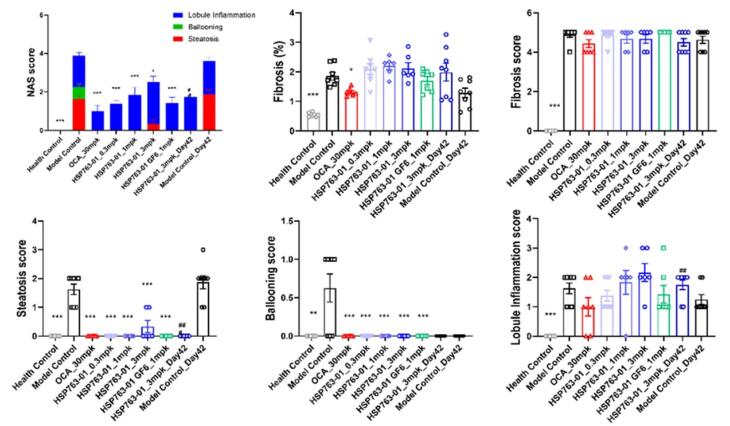
Change in NAFLD activity score, fibrosis score, steatosis score, ballooning score, lobule inflammation score and change in fibrosis areas (health control compared to Model control or post-treatment) for individual animals in the different treatment groups: Data shown were from lean control mice, DIO-NASH vehicle controls (Vehicle PO) and DIO-NASH mice treated with OCA or HSP763-01. Data were presented as mean ± SEM. *** P < 0.001, ** P < 0.01, * P < 0.05 vs NASH Control, analyzed by one-way ANOVA. Health control vs NASH control, analyzed by t-test ###* P* < 0.001, ##* P* < 0.01, #*p* < 0.05 vs model control Day 42, analyzed by t-test

 At present, there are two evaluation systems for the pathological diagnosis of NASH, one is the NAS scoring system proposed by the American Association of Hepatology,^42^ and the other is the SAF scoring system proposed by the European Association of Hepatology.^43,44^ Pathologists score sections according to the criteria shown in Table S3 and Table S4 above. In various dosage groups, significant reductions in liver steatosis and ballooning scores were observed compared to the corresponding model control group (*P* < 0.001). However, the efficacy of reducing lobule inflammation was inferior to that of the positive control OCA group, with substantial individual variations and significant fluctuations among different dosage groups. Regarding liver fibrosis levels, due to individual differences, there was no discernible trend of alleviation compared to the positive control OCA group, similar to the model control group. This may be attributed to severe CCl_4_-induced liver fibrosis (grade 5), which was challenging to reverse within a short timeframe. The analysis as shown in Figure 7.

**Figure 8:**
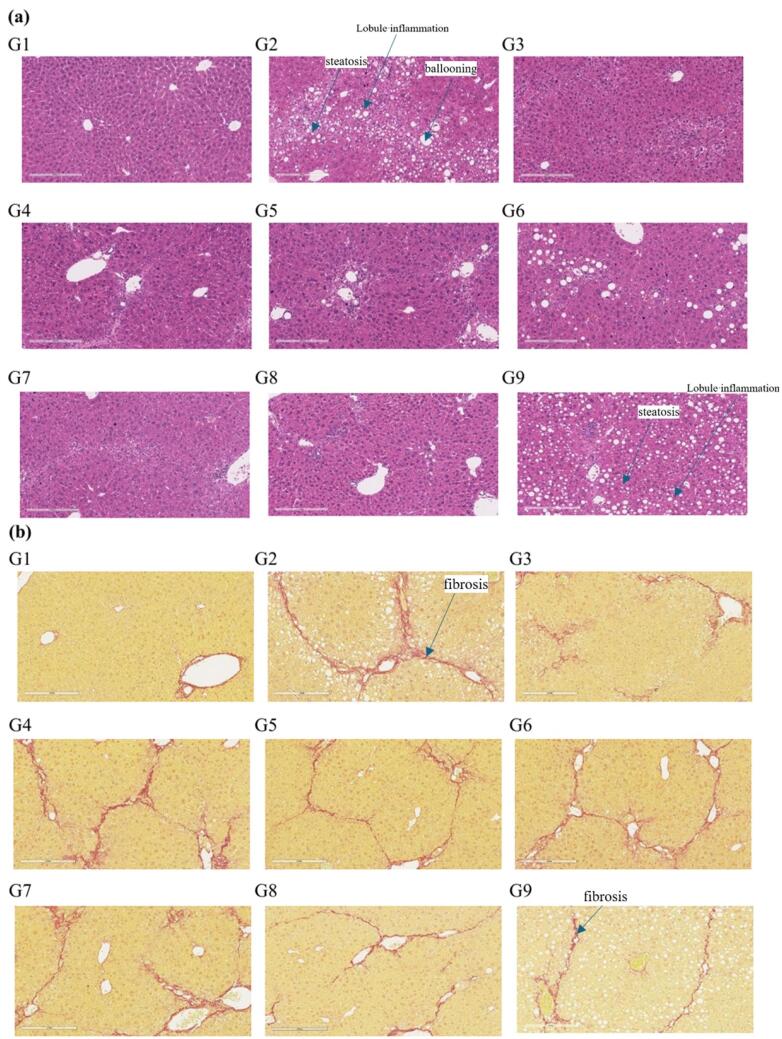
Representative images of liver morphology at termination of the study. H&E staining (20 × , scale bar = 100 μm). **(b)** Sirius red staining (20 × , scale bar = 100 μm). G1 (healthy control group), G2 (model control), G3 (OCA positive group), G4 (0.3 mpk), G5 (1 mpk), G6 (3 mpk), G7 (1 mpk, stored at -20 °C for 30M), G8 (3 mpk Day 42), G9 (model control Day 42)

 The NAS score was evaluated by HE staining and the morphological changes in the liver and the significant improvement in histopathological NAS score (given group vs. corresponding model group) were observed (Figure 8a). The changes in fibrosis as detected by Sirus-red staining in the tissues (given group vs. corresponding model group, Figure 8b). From the histopathological results of the liver, compared with the model group, HSP763-01 at all doses significantly reduced the NAS Score level. However, compared to the model group, the OCA positive control showed a certain reversal effect on bridging fibrosis.^22^ In the HSP763-01 treatment group, only the medium and high doses showed a minor effect on fibrosis relief and there were no significant difference. In summary, HSP763-01 showed certain improvement effects in the HFD + CCl_4_ model.

## Discussion

 The pathological hallmark of NASH liver injury is an increase in the accumulation of lipid droplets within liver cells, which primarily manifests as the air-filled and vacuolated degeneration of hepatocytes due to fat accumulation within the cells, leading to hepatocyte apoptosis, infiltration of inflammatory factors, and ultimately, patchy and focal hepatocyte death. The onset of NASH revolves around fatty liver injury, and fatty degeneration, lobular inflammation, ballooning of hepatocytes, and fibrosis are considered its histological features.^4,45^ Due to the synergistic effect of FGF21 and GLP-1, this study primarily combined the GLP-1 target and FGF21 target on a dual action basis. Additionally, point mutations were made on the amino acid sequence of the humanized FGF21 protein to construct a novel GLP-1-Fc-FGF21 fusion protein. The activation of the GLP-1 receptor stimulates insulin secretion while inhibiting glucagon release, reduces appetite and enhances satiety in the brain, regulates lipid metabolism, improves insulin sensitivity, and exerts anti-inflammatory effects. By activating AMPK to mitigate hepatic fat accumulation, AMPK inhibits fatty acid synthase and promotes beta-oxidation of fatty acids, thereby decreasing lipid droplet accumulation within hepatocytes; concurrently, it suppresses the NF-κB pathway and diminishes the release of pro-inflammatory cytokines (such as TNF-α and IL-6), thus attenuating inflammatory pathways. Furthermore, activation of the PI 3K/Akt signaling pathway reduces cellular apoptosis and aids in preventing NASH from advancing to liver fibrosis, potentially facilitating a reversal of liver fibrosis.^46,47^ In alignment with its direct anti-inflammatory effects across various cell types, FGF-21 promotes NRF-2 nuclear translocation to inhibit the expression of pro-inflammatory cytokines TNF-α, IL-6, IL-1β, and IFN-γ in macrophages, thereby obstructing NF-κB activation.^48^ The anti-inflammatory properties of FGF-21 may arise from its direct suppression of pro-inflammatory signal transduction pathways in both innate and potentially adaptive immune cell populations, as well as a reduction in the infiltration of immune effector cells into the liver. Additionally, it exerts indirect inhibition on pro-inflammatory signals released by damaged hepatocytes. Furthermore, FGF-21 mitigates fibrosis in a chemical-induced liver injury mouse model by inhibiting TGF-β expression and signaling alongside NF-κB activation.^16,49^ Notably, FGF-21 demonstrates potent triglyceride-lowering effects through the acceleration of lipolysis and metabolism within white and brown adipose tissues rather than via direct signaling to adipose tissue.^49,50^ Consequently, based on the distinct mechanisms of action of GLP-1 and FGF21, these two factors exhibit a synergistic effect on lipid metabolism, inflammation suppression, hepatic protection, and the attenuation of fibrosis progression. OCA acts as a highly specific FXR agonist, regulating bile acid synthesis and releasing fibroblast growth factor-19 (FGF-19) into the portal vein circulation to modulate hepatic triglyceride synthesis, fibrosis, and other metabolic pathways in hepatocytes.^51^ It selectively binds to and activates FXR in intestinal and liver epithelial cells, thereby reducing toxic bile acid levels, effectively mitigating the detrimental effects of bile acids on liver tissue.^52,53^ Additionally, it inhibits liver triglyceride synthesis, promotes insulin sensitivity and function, and reduces the occurrence and progression of NASH. The most commonly reported adverse events associated with OCA treatment include pruritus, fatigue, abdominal pain, and discomfort. Pruritus incidence is dose-dependent and higher when OCA is used as monotherapy.^53,54^ HSP763-01 functions as a dual-target fusion protein of GLP-1 and FGF21 to prevent the common adverse reactions caused by OCA. To verify the efficacy of the fusion protein, we established an HFD/CCl_4_ mouse model. High-fat diet (HFD) induced an increase in body weight and liver weight in mice compared to mice fed with normal diet. The levels of TC, HDL-C, and LDL-C in the serum of DIO mice were significantly increased, which simulated the symptoms of NASH. The combination of DIO mice and CCl_4_ accelerated and worsened the pathological process of the NASH mouse model. The levels of ALT and AST in the serum of HFD + CCl_4_ group were significantly higher than those of HFD group, and CCl_4_ induced further liver damage, shortened the induction time, and aggravated liver fibrosis in DIO mice.^55-57^

 Compared with the model control group, the low, medium, and high dose groups given twice a week for 15 days showed a rapid weight loss to normal levels within 15 days, which was significantly different from the positive control group OCA and showed a dose-dependent trend. After continued administration, the weight loss was not significant and maintained at normal levels, indicating that the recombinant protein HSP763-01 has no further trend of lowering normal weight; the high dose group given once a week was slower in weight loss than the low and medium dose groups given twice a week, and the weight was reduced to normal level by 24 days. Compared with the model control group, the weight of liver and adipose tissue in the treatment group was significantly lower, which was significantly different from the positive control group OCA, indicating that HSP763-01 has the function of reducing fat synthesis and promoting fat metabolism utilization in vivo, and can be used for the treatment of obesity and metabolic syndrome caused by obesity. From the blood biochemical results, compared with the model group, HSP763-01 had a significant lowering effect on TG, TCHO, LDL-C, and FFA levels, especially on TG, TCHO, and FFA levels in the liver of NASH model mice. The presence of TG in the bloodstream is often associated with hepatic accumulation of TG. In mouse models of NASH, hepatic steatosis and inflammation can disrupt lipid metabolism, leading to increased release of TG from the liver into the bloodstream, resulting in elevated levels of circulating TG. Cholesterol levels in the blood are also influenced by metabolic disorders in the liver. The liver plays a crucial role in cholesterol metabolism, and impaired liver function can affect synthesis, transport, and clearance of cholesterol from the bloodstream. This may lead to an increase in LDL cholesterol levels and a relative decrease in HDL cholesterol levels. Following administration of HSP763-01, there was a significant reduction (P < 0.001) observed in both hepatic TG and TCHO content in mice, showing a certain linear correlation between them. Due to this substantial decrease in TG levels within the liver, hydrolysis of TG resulted in a large amount of FFA, which were released into the bloodstream but did not show significant changes overall. However, FFA content within the liver significantly increased indicating delayed release into circulation at Day 28. With prolonged administration time (Day 42), FFA content within the liver further decreased (*P* < 0.05). Notably, there were remarkable improvements observed regarding hepatic steatosis, inflammation as well as balloon-like lesions (*P* < 0.001), gradually restoring surface liver function. It is important to highlight that analyse of liver tissue, biochemical indicators in mouse serum and liver weight indicated no significant differences between the high-dose group (3 mpk) and the medium-dose group (1 mpk) (*P* > 0.05). This finding suggests that the medium-dose group (1 mpk) has reached a plateau in therapeutic efficacy, as further increases in dosage do not result in significant reductions. the medium-dose group (1 mpk) achieved levels comparable to those observed in normal mice. Based on the potential synergistic mechanisms of GLP-1 and FGF21 targets, there appears to be no discernible therapeutic effect on normal mice. However, blood glucose levels were significantly reduced to within the healthy range (*P* < 0.001), and even lower, in all dosage groups, demonstrating a clear dose-dependent effect. Due to the lipid metabolism disorder in NASH model mice, HDL-C levels are abnormally high. HSP763-01 could lower HDL-C levels and restore them to normal levels. Based on the liver pathological results, it was observed that HSP763-01 at all doses significantly reduced the NAS Score level compared to the model group. This reduction was particularly evident in alleviating fatty degeneration and ballooning degeneration, demonstrating a clear dose-dependent relationship. However, there was no anticipated reduction in lobular inflammation scores; individual variations were substantial, and dosage appeared to have an opposite effect. There are several reasons why LDL-C, leaflet inflammation, and fibrosis exhibit significant individual variability in the study, Firstly, Genes can influence lipid metabolism, inflammatory responses, and the development of fibrosis.^58^ For example, some genes may affect the way the liver processes LDL-C, either promoting or inhibiting its uptake and clearance. Variations in genes related to the immune system can lead to differences in the intensity and nature of leaflet inflammation.^59^ Secondly, Even within a controlled experimental setting, there can be slight differences in the diet intake of individual mice, Even with the same dose of the HSP763-01, there were differences that led to inflammation and the development of liver fibrosis in individual mice. Thirdly, Microbiome differences, the gut microbiota of each mouse is unique, certain bacteria in the gut can influence the metabolism of lipids, including LDL-C.^60^ In conclusion, a complex interplay of genetic, dietary/environmental, microbiome, and stochastic factors significantly contributes to the individual variability observed in LDL-C levels, leaflet inflammation, and fibrosis following treatment with HSP760-01 in the NASH mouse model. Based on the fibrosis scoring results, HSP763-01 showed a slight reduction in fibrosis score, although it did not reach statistical significance and was less effective in alleviating liver fibrosis compared to the positive control OCA. This may be attributed to the advanced stage of fibrosis induced by CCl_4_ in the mouse model (grade 3), which is challenging to reverse within a short timeframe. CCl_4_ induces rapid and extensive liver damage and fibrosis. The liver’s normal architecture is quickly disrupted as hepatocytes are damaged, leading to the activation of stellate cells which then produce excessive extracellular matrix components like collagen, causing fibrosis. In a short time, the fibrotic changes can become quite severe, making it difficult to reverse quickly. There are numerous complex cellular and molecular alterations that occur during CCl_4_ induced fibrosis. For example, there are changes in the balance of pro-fibrotic and anti-fibrotic cytokines, growth factors, and signaling pathways.^50^ Reversing these changes simultaneously within a short period is challenging as they are intertwined and affect multiple cell types in the liver. As the fibrosis progresses to a severe stage due to CCl_4_ toxicity, the liver’s natural regenerative ability is hampered. The fibrotic tissue can form barriers that prevent proper communication between cells and impede the access of progenitor cells to the damaged areas, making it hard for the liver to repair itself in a short time. Longer treatment durations can potentially address some of these limitations. With more time, it may be possible to gradually correct the imbalances in cytokines and signaling pathways. CCl_4_-induced liver fibrosis may be related with multi biological process, pathway and targets which may provide potential protection reaction mechanism for CCl_4_ detoxication in the liver.^61^ Also, longer treatment might give the liver cells more opportunity to regenerate and replace the fibrotic tissue, especially if combined with strategies to enhance liver cell regeneration like using growth factors or stem cell-based therapies.

## Conclusion

 HSP763-01 demonstrated significant dual-targeting efficacy for GLP-1 and FGF21, leading to notable improvements in lipid metabolism, blood glucose control, and weight reduction in the HFD/CCl4 mouse model. The compound also showed substantial alleviation of liver steatosis and ballooning, with partial effects on reducing lobular inflammation and fibrosis. To further assess the therapeutic potential of HSP763-01, future studies should consider models that induce progressive, mild fibrosis over a longer duration, such as the AMLN diet^62^ or genetically modified models that more closely mimic human NASH pathology (e.g., CETP deficiency).^63-65^ Additionally, evaluating HSP763-01’s pharmacokinetic profile in mice will be essential to optimize dosing strategies for sustained therapeutic effects.

## Competing Interests

 The authors declare no conflict of interest.

## Ethical Approval

 All assessments were conducted in accordance with ethical principles and under the supervision of WuXi AppTec’s institutional Animal Care and Use Committee (IACUC) (Ethic NO. GP01-QD021-2021).

## Supplementary Files

Supplementary file 1 contains Tables S1-S4.

## References

[1] Younossi ZM, Koenig AB, Abdelatif D, Fazel Y, Henry L, Wymer M (2016). Global epidemiology of nonalcoholic fatty liver disease-meta-analytic assessment of prevalence, incidence, and outcomes. Hepatology.

[2] Sheka AC, Adeyi O, Thompson J, Hameed B, Crawford PA, Ikramuddin S (2020). Nonalcoholic steatohepatitis: a review. JAMA.

[3] Younossi ZM, Golabi P, Paik JM, Henry A, Van Dongen C, Henry L (2023). The global epidemiology of nonalcoholic fatty liver disease (NAFLD) and nonalcoholic steatohepatitis (NASH): a systematic review. Hepatology.

[4] Friedman SL, Neuschwander-Tetri BA, Rinella M, Sanyal AJ (2018). Mechanisms of NAFLD development and therapeutic strategies. Nat Med.

[5] Perumpail BJ, Khan MA, Yoo ER, Cholankeril G, Kim D, Ahmed A (2017). Clinical epidemiology and disease burden of nonalcoholic fatty liver disease. World J Gastroenterol.

[6] Petta S, Targher G, Romeo S, Pajvani UB, Zheng MH, Aghemo A (2024). The first MASH drug therapy on the horizon: Current perspectives of resmetirom. Liver Int.

[7] Food and Drug Administration (FDA). FDA Approves First Treatment for Patients with Liver Scarring Due to Fatty Liver Disease. FDA; 2024. Available from: https://www.fda.gov/news-events/press-announcements/fda-approves-first-treatment-patients-liver-scarring-due-fatty-liver-disease.

[8] Caddeo A, Romeo S (2025). Precision medicine and nucleotide-based therapeutics to treat steatotic liver disease. Clin Mol Hepatol.

[9] Bittla P, Paidimarri SP, Ayuthu S, Chauhan YD, Saad MZ, Mirza AA (2024). Resmetirom: a systematic review of the revolutionizing approach to non-alcoholic steatohepatitis treatment focusing on efficacy, safety, cost-effectiveness, and impact on quality of life. Cureus.

[10] Wang JY, Wang QW, Yang XY, Yang W, Li DR, Jin JY (2023). GLP-1 receptor agonists for the treatment of obesity: role as a promising approach. Front Endocrinol (Lausanne).

[11] Drucker DJ (2018). Mechanisms of action and therapeutic application of glucagon-like peptide-1. Cell Metab.

[12] Holst JJ (2007). The physiology of glucagon-like peptide 1. Physiol Rev.

[13] Trujillo JM, Nuffer W, Smith BA (2021). GLP-1 receptor agonists: an updated review of head-to-head clinical studies. Ther Adv Endocrinol Metab.

[14] Wang Z, Sun T, Yu J, Li S, Gong L, Zhang Y (2023). FGF21: a sharp weapon in the process of exercise to improve NAFLD. Front Biosci (Landmark Ed).

[15] Sonoda J, Chen MZ, Baruch A. FGF21-receptor agonists: an emerging therapeutic class for obesity-related diseases. Horm Mol Biol Clin Investig 2017;30(2). doi: 10.1515/hmbci-2017-0002. 28525362

[16] Tillman EJ, Rolph T (2020). FGF21: an emerging therapeutic target for non-alcoholic steatohepatitis and related metabolic diseases. Front Endocrinol (Lausanne).

[17] Weng Y, Ishino T, Sievers A, Talukdar S, Chabot JR, Tam A (2018). Glyco-engineered long acting FGF21 variant with optimal pharmaceutical and pharmacokinetic properties to enable weekly to twice monthly subcutaneous dosing. Sci Rep.

[18] Hameed B, Terrault NA, Gill RM, Loomba R, Chalasani N, Hoofnagle JH (2018). Clinical and metabolic effects associated with weight changes and obeticholic acid in non-alcoholic steatohepatitis. Aliment PharmacolTher.

[19] Haczeyni F, Poekes L, Wang H, Mridha AR, Barn V, Geoffrey Haigh W (2017). Obeticholic acid improves adipose morphometry and inflammation and reduces steatosis in dietary but not metabolic obesity in mice. Obesity (Silver Spring).

[20] Siddiqui MS, Van Natta ML, Connelly MA, Vuppalanchi R, Neuschwander-Tetri BA, Tonascia J (2020). Impact of obeticholic acid on the lipoprotein profile in patients with non-alcoholic steatohepatitis. J Hepatol.

[21] Jouihan H, Will S, Guionaud S, Boland ML, Oldham S, Ravn P (2017). Superior reductions in hepatic steatosis and fibrosis with co-administration of a glucagon-like peptide-1 receptor agonist and obeticholic acid in mice. Mol Metab.

[22] Chalasani N, Abdelmalek MF, Loomba R, Kowdley KV, McCullough AJ, Dasarathy S (2019). Relationship between three commonly used non-invasive fibrosis biomarkers and improvement in fibrosis stage in patients with non-alcoholic steatohepatitis. Liver Int.

[23] Gilroy CA, Capozzi ME, Varanko AK, Tong J, D’Alessio DA, Campbell JE (2020). Sustained release of a GLP-1 and FGF21 dual agonist from an injectable depot protects mice from obesity and hyperglycemia. Sci Adv.

[24] Yadav P, Khurana A, Bhatti JS, Weiskirchen R, Navik U (2022). Glucagon-like peptide 1 and fibroblast growth factor-21 in non-alcoholic steatohepatitis: an experimental to clinical perspective. Pharmacol Res.

[25] Milani I, Codini M, Guarisco G, Chinucci M, Gaita C, Leonetti F (2024). Hepatokines and MASLD: the GLP1-RAs-FGF21-fetuin-A crosstalk as a therapeutic target. Int J Mol Sci.

[26] Gilroy CA, Capozzi M, Su JC, Tong J, D’Alessio DA, Campbell J (2019). A novel GLP-1 and FGF-21 dual agonist drug protects mice from obesity and hyperglycemia. Diabetes.

[27] Pan Q, Lin S, Li Y, Liu L, Li X, Gao X (2021). A novel GLP-1 and FGF21 dual agonist has therapeutic potential for diabetes and non-alcoholic steatohepatitis. EBioMedicine.

[28] Araujo LC, Dias CC, Sucupira FG, Ramalho LN, Camporez JP. A short-term rodent model for non-alcoholic steatohepatitis induced by a high-fat diet and carbon tetrachloride. Biosci Rep 2024;44(5). doi: 10.1042/bsr20231532. PMC11081943 38660995

[29] Sun F, Ren J (2023). Advances in the diagnosis of nonalcoholic fatty liver disease. Chin Bull Life Sci.

[30] Scheen AJ (2016). Dulaglutide (LY-2189265) for the treatment of type 2 diabetes. Expert Rev Clin Pharmacol.

[31] Glaesner W, Mark Vick A, Millican R, Ellis B, Tschang SH, Tian Y (2010). Engineering and characterization of the long‐acting glucagon‐like peptide‐1 analogue LY2189265, an Fc fusion protein. Diabetes Metab Res Rev.

[32] Gurung T, Shyangdan DS, O’Hare JP, Waugh N (2015). A novel, long-acting glucagon-like peptide receptor-agonist: dulaglutide. Diabetes MetabSyndrObes.

[33] Skrivanek Z, Berry S, Berry D, Chien J, Geiger MJ, Anderson JH (2012). Application of adaptive design methodology in development of a long-acting glucagon-like peptide-1 analog (dulaglutide): statistical design and simulations. J Diabetes Sci Technol.

[34] Jimenez-Solem E, Rasmussen MH, Christensen M, Knop FK (2010). Dulaglutide, a long-acting GLP-1 analog fused with an Fc antibody fragment for the potential treatment of type 2 diabetes. CurrOpin Mol Ther.

[35] Uccellatore A, Genovese S, Dicembrini I, Mannucci E, Ceriello A (2015). Comparison review of short-acting and long-acting glucagon-like peptide-1 receptor agonists. Diabetes Ther.

[36] Kinne AS, Tillman EJ, Abdeen SJ, Johnson DE, Parmer ES, Hurst JP (2023). Noncompetitive immunoassay optimized for pharmacokinetic assessments of biologically active efruxifermin. J Pharm Biomed Anal.

[37] Raptis DD, Mantzoros CS, Polyzos SA (2023). Fibroblast growth factor-21 as a potential therapeutic target of nonalcoholic fatty liver disease. Ther Clin Risk Manag.

[38] Puengel T, Tacke F (2023). Efruxifermin, an investigational treatment for fibrotic or cirrhotic nonalcoholic steatohepatitis (NASH). Expert OpinInvestig Drugs.

[39] Kaufman A, Abuqayyas L, Denney WS, Tillman EJ, Rolph T (2020). AKR-001, an Fc-FGF21 analog, showed sustained pharmacodynamic effects on insulin sensitivity and lipid metabolism in type 2 diabetes patients. Cell Rep Med.

[40] Li J, Ge QY, Song QY, Zhang ZH (2023). [Research progress on the histological scoring system for nonalcoholic fatty liver disease]. Zhonghua Gan Zang Bing Za Zhi.

[41] Brunt EM, Kleiner DE, Wilson LA, Belt P, Neuschwander-Tetri BA (2011). Nonalcoholic fatty liver disease (NAFLD) activity score and the histopathologic diagnosis in NAFLD: distinct clinicopathologic meanings. Hepatology.

[42] Kleiner DE, Brunt EM, Van Natta M, Behling C, Contos MJ, Cummings OW (2005). Design and validation of a histological scoring system for nonalcoholic fatty liver disease. Hepatology.

[43] Bedossa P, Poitou C, Veyrie N, Bouillot JL, Basdevant A, Paradis V (2012). Histopathological algorithm and scoring system for evaluation of liver lesions in morbidly obese patients. Hepatology.

[44] Bedossa P (2014). Utility and appropriateness of the fatty liver inhibition of progression (FLIP) algorithm and steatosis, activity, and fibrosis (SAF) score in the evaluation of biopsies of nonalcoholic fatty liver disease. Hepatology.

[45] Huby T, Gautier EL (2022). Immune cell-mediated features of non-alcoholic steatohepatitis. Nat Rev Immunol.

[46] Smith NK, Hackett TA, Galli A, Flynn CR (2019). GLP-1: molecular mechanisms and outcomes of a complex signaling system. Neurochem Int.

[47] Zheng Z, Zong Y, Ma Y, Tian Y, Pang Y, Zhang C (2024). Glucagon-like peptide-1 receptor: mechanisms and advances in therapy. Signal Transduct Target Ther.

[48] Adams AC, Coskun T, Rovira AR, Schneider MA, Raches DW, Micanovic R (2012). Fundamentals of FGF19 & FGF21 action in vitro and in vivo. PLoS One.

[49] BonDurant LD, Ameka M, Naber MC, Markan KR, Idiga SO, Acevedo MR, et al. FGF21 regulates metabolism through adipose-dependent and -independent mechanisms. Cell Metab 2017;25(4):935-44.e4. doi: 10.1016/j.cmet.2017.03.005. PMC5494834 28380381

[50] Jia MQ, Guan CX, Tao JH, Zhou Y (2022). Research progress of fibroblast growth factor 21 in fibrotic diseases. Oxid Med Cell Longev.

[51] Levy C, Manns M, Hirschfield G (2023). New treatment paradigms in primary biliary cholangitis. Clin Gastroenterol Hepatol.

[52] Mudaliar S, Henry RR, Sanyal AJ, Morrow L, Marschall HU, Kipnes M, et al. Efficacy and safety of the farnesoid X receptor agonist obeticholic acid in patients with type 2 diabetes and nonalcoholic fatty liver disease. Gastroenterology 2013;145(3):574-82.e1. doi: 10.1053/j.gastro.2013.05.042. 23727264

[53] Mousa HS, Lleo A, Invernizzi P, Bowlus CL, Gershwin ME (2015). Advances in pharmacotherapy for primary biliary cirrhosis. Expert OpinPharmacother.

[54] LiverTox: Clinical and Research Information on Drug-Induced Liver Injury. Bethesda (MD): National Institute of Diabetes and Digestive and Kidney Diseases; 2019. 31643176

[55] Liao N, Zheng Y, Xie H, Zhao B, Zeng Y, Liu X (2017). Adipose tissue-derived stem cells ameliorate hyperglycemia, insulin resistance and liver fibrosis in the type 2 diabetic rats. Stem Cell Res Ther.

[56] Meng Q, Li X, Xiong X (2022). Identification of hub genes associated with non-alcoholic steatohepatitis using integrated bioinformatics analysis. Front Genet.

[57] Wang XX, Jin R, Li XH, Yang Q, Teng X, Liu FF (2023). Collagen co-localized with macrovesicular steatosis better differentiates fibrosis progression in non-alcoholic fatty liver disease mouse models. Front Med (Lausanne).

[58] Marques SM, Campos PP, Castro PR, Cardoso CC, Ferreira MA, Andrade SP (2011). Genetic background determines mouse strain differences in inflammatory angiogenesis. Microvasc Res.

[59] Walkin L, Herrick SE, Summers A, Brenchley PE, Hoff CM, Korstanje R (2013). The role of mouse strain differences in the susceptibility to fibrosis: a systematic review. Fibrogenesis Tissue Repair.

[60] Schoeler M, Caesar R (2019). Dietary lipids, gut microbiota and lipid metabolism. Rev EndocrMetabDisord.

[61] Dong S, Chen QL, Song YN, Sun Y, Wei B, Li XY (2016). Mechanisms of CCl4-induced liver fibrosis with combined transcriptomic and proteomic analysis. J Toxicol Sci.

[62] Gwag T, Reddy Mooli RG, Li D, Lee S, Lee EY, Wang S (2021). Macrophage-derived thrombospondin 1 promotes obesity-associated non-alcoholic fatty liver disease. JHEP Rep.

[63] Larter CZ, Yeh MM, Haigh WG, Van Rooyen DM, Brooling J, Heydet D (2013). Dietary modification dampens liver inflammation and fibrosis in obesity-related fatty liver disease. Obesity (Silver Spring).

[64] Jahn D, Kircher S, Hermanns HM, Geier A (2019). Animal models of NAFLD from a hepatologist’s point of view. BiochimBiophys Acta Mol Basis Dis.

[65] Peng C, Stewart AG, Woodman OL, Ritchie RH, Qin CX (2020). Non-alcoholic steatohepatitis: a review of its mechanism, models and medical treatments. Front Pharmacol.

