# Supplementary material for: A Novel GLP-1 and FGF21 Fusion Protein for the Treatment of Non-alcoholic Steatohepatitis (NASH)
Source: Adv Pharm Bull. 2024 Dec 10;15(1):162–75. doi: 10.34172/apb.43672 (PMC12235361; doi:10.34172/apb.43672)
Supplement: Supplementary file 1 — contains Tables S1-S4. [file apb-15-162-s001.pdf]

***Adv Pharm Bull*.** 2025;15(1):162-S1

doi: 10.34172/apb.43672

https://apb.tbzmed.ac.ir

Supplementary file 1

Table 1. Detailed administration protocol

Group	Number of animals per group	CCl4	Forage	Administration and treatment of each group	Note
**1**	8	No	Normal	Vehicle, s.c. B.i.w., Day 0~Day 27	Blank control model
**2**	8	Yes	High-fat	Vehicle, s.c. CCl4, i.p. B.i.w., Day 0~Day 27	As a control group for models within groups 3-7
**3**	8	Yes	High-fat	OCA, 30 mg/kg, p.o., q.d., Day 0~Day 27	The CCl4 model induction and administration were conducted simultaneously, CCl4, i.p. B.i.w; the batch HSP763-01 GF6 was stored at -20°C for more than 30 M, while the batch HSP763-01 was stored at -20°C for less than 1 M.
**4**	8	Yes	High-fat	HSP763-01, 0.3 mg/kg, s.c. B.i.w ., Day 0~Day 27	
**5**	8	Yes	High-fat	HSP763-01,1.0mg/kg, s.c. B.i.w ., Day 0~Day 27	
**6**	8	Yes	High-fat	HSP763-01, 3.0 mg/kg, s.c. B.i.w ., Day 0~Day 27	
**7**	8	Yes	High-fat	HSP763-01 GF6, 1.0mg/kg, s.c. B.i.w ., Day 0~Day 27	
**8**	8	Yes	High-	HSP763-01, 3.0mg/kg, s.c.	The pre-treatment period involved the use of CCl4 for 2 weeks, followed by a 4 weeks drug

			fat	B.i.w ., Day 14~Day 41 CCl4, i.p. once a week, Day 0~Day 41	administration
**9**	8	Yes	High-fat	CCl4, i.p. once a week, Day 0~Day 41	As a model comparison for Group8, CCl4 continued to induce to Day 41 based on Group2.

Table 2. SEC-HPLC

**Name**	**Instruction**	
Mobile phase	100mM PB,200mM Arg, pH 6.8	
Chromatography column	Waters BEH200	
High-Performance Liquid Chromatograph (HPLC)	Agilent 1260 InfinityII	
**Procedure**	**Instruction**	**Parameter**
Sample preparation	Take a protein sample and place it in the liquid phase inner tube, then directly load it onto the column.	
Chromatographic parameters	Loading	50ug
	Flow velocity	1ml/min
	Collection time	15min
	Detection wavelength	280nm
Operating Instructions	Stabilize the baseline of the system by equilibrating it with the mobile phase. Subsequently, add 50 µg of antibody to the designated sample vial and place the vials within the instrument. Collect data for a duration of 15 minutes, followed by the analysis and processing of the data. Ensure that the data is securely saved.	

Table 3. NAS Assessment

NASH			
Pathological manifestations	Assessment	Scoring (NAS）	Assessment Criteria for Pathological Changes：
Hepatocyte ballooning	None	0	1. Hepatocyte ballooning: Pathological changes similar to air bubbles are observed in hepatocytes. Due to the liquid vacuole-like changes, the size of hepatocytes increases, and the nucleus of the hepatocyte is concentrated or displaced.<br>2. Inflammatory cell infiltration: Large numbers of inflammatory cells, mainly neutrophils and macrophages, are found in the portal area, subcapsular vein area, or around the liver lobules.<br>3. Changes in liver cell fat: regular round vacuoles are observed in liver cells of different sizes, with the nucleus located at the edge.
	A few balloon-shaped cells	1	
	Large numbers of balloon-shaped cells	2	
Overall assessment of small follicular inflammation in all inflammatory lesions.	None	0	
	< 2 foci/200-fold field of view	1	
	2-4 foci/ 200-fold field of view	2	
	>4 foci / 200-fold field of view	3	
Lipid degeneration	<5%	0	
	5%-33%	1	
	>33%-66%	2	
	>66%	3	

Table 4. Staging criteria for liver fibrosis

<table>
<tr><th colspan="10">Hepatic Fibrosis</th></tr>
<tr><td>Histopathological characteristics</td><td colspan="9">Staging criteria for liver fibrosis</td></tr>
<tr><td rowspan="4">Fibrosis Score</td><td rowspan="2">Staging</td><td rowspan="2">0</td><td colspan="3">1</td><td rowspan="2">2</td><td rowspan="2">3</td><td rowspan="2">4</td></tr>
<tr><td>1A</td><td>1B</td><td>1C</td></tr>
<tr><td>Scoring</td><td>0</td><td>1</td><td>2</td><td>3</td><td>4</td><td>5</td><td>6</td></tr>
<tr><td>Categorical definition</td><td>None</td><td>Mild, Zone 3, peritoneal fibrosis.</td><td>Moderate, Zone 3, Peripheral Fibrosis</td><td>Portal area / Periportal fibrosis</td><td>Sinusoid/portal area / Perivascular fibrosis</td><td>Bridging fibrosis</td><td>Hepatic cirrhosis</td></tr>
</table>
